# ArthroRad trial: multicentric prospective and randomized single-blinded trial on the effect of low-dose radiotherapy for painful osteoarthritis depending on the dose—results after 3 months’ follow-up

**DOI:** 10.1007/s00066-021-01866-2

**Published:** 2021-11-01

**Authors:** Marcus Niewald, Lara Natalie Müller, Matthias G. Hautmann, Yvonne Dzierma, Patrick Melchior, Stefan Gräber, Christian Rübe, Jochen Fleckenstein, working group “Benign diseases” of the DEGRO (German Society for Radiation Oncology)

**Affiliations:** 1 Dept. of Radiotherapy and Radiooncology, Saarland University Medial Center, Kirrberger Str. 100, Building. 6.5, 66424 Homburg/Saar, Germany; 2 Dept. of Radiotherapy, University Hospital, Regensburg, Germany; 3 Institute for Medical Biometry, Epidemiology and Medical Informatics, Saarland University Medical Center, Homburg/Saar, Germany

**Keywords:** Osteoarthritis, Radiotherapy, Low dose, Pain relief, Quality of life

## Abstract

**Purpose:**

Randomized comparison of the effect of radiotherapy on painful osteoarthritis (OA) applying a standard-dose vs. a very-low-dose regime

**Patients and methods:**

Patients with OA of the hand and knee joints were included. Further inclusion criteria: symptoms for more than 3 months, favorable general health status, age above 40 years. Patients with prior local radiotherapy, trauma, rheumatoid arthritis, or vascular diseases were excluded. After randomization (every joint was randomized separately), the following protocols were applied: standard arm: total dose 3.0 Gy, single fractions of 0.5 Gy twice weekly; experimental arm: total dose 0.3 Gy, single fractions of 0.05 Gy twice weekly. The dosage was not known to the patients. The patients were examined 3 and 12 months after radiotherapy. Scores like VAS (visual analogue scale), KOOS-SF (the knee injugy and osteoarthritis outcome score), SF-SACRAH (short form score for the assessment and quantification of chronic rheumatic affections of the hands), and SF-12 (short form 12) were used.

**Results:**

A total of 64 knees and 172 hands were randomized. 3.0 Gy was applied to 87 hands and 34 knees, 0.3 Gy was given to 85 hands and 30 knees. After 3 months, we observed good pain relief after 3 Gy and after 0.3 Gy, there was no statistically significant difference. Side effects were not recorded. The trial was closed prematurely due to slow recruitment.

**Conclusion:**

We found favorable pain relief and a limited response in the functional and quality of life scores in both arms. The effect of low doses such as 0.3 Gy on pain is widely unknown. Further trials are necessary to compare a conventional dose to placebo and to further explore the effect of low doses on inflammatory disorders.

## Background

Osteoarthritis is a very frequent disease, especially in elderly people. Caused by overweight, improper loading of the joint, injuries, dysplasia, arthritis, or other arthropathies, progressive destruction of the joint cartilage may potentially involve the bone, the joint capsule, and the adjacent muscles [1, 2]. Very frequently, OA is a cause of pain. In the beginning, only repeated movements or burden applied to the joint are painful. Later, pain may occur during rest or at night. The joints are deformed, passive and/or active mobility are impaired.

Conservative treatment methods involve weight reduction, physiotherapy, and orthopedic devices. Local and oral analgesics are often prescribed. More invasively, corticoids and hyaluronic acid are injected into the joints. In case of severe synovitis, radiosynoviorthesis may be recommendable. Finally, joint-preserving or joint-replacing surgical interventions are performed [3, 4].

The analgesic effect of radiotherapy in patients with OA has been known for a long time. There is a large body of retrospective publications showing a good analgesic effect of radiotherapy for osteoarthritis of the knee joint in 58–91% of patients [2], whereas literature on hand and finger joints is rare [1].

There have been ample research activities on arthritis models in order to clarify the mechanism of the effect of radiotherapy in OA treatment, which have led to an improved understanding. Radiation has been shown to inhibit the adhesion of macrophages to the endothelium, induce expression of the x‑linked apoptosis inhibitor, of TGFß, reduce the expression of E‑ and L‑selectin, and inhibit the expression of IL‑1 and CCL 20. All these effects are maximal after single doses of 0.3–0.7 Gy [5–7].

We thus conducted a prospective randomized trial in order to examine the effect of radiotherapy on painful OA and to achieve a high level of evidence.

## Patients and methods

Patients meeting the following criteria were included into this trial: clinical diagnosis of OA of the knee and/or hand or finger joints, radiological proof of the diagnosis (plain radiographs), duration of anamnesis more than 3 months, favorable general health status.

Patients presenting with previous joint replacement; previous radiation therapy to the affected joint; previous trauma; rheumatic, arterial, or venous vessel diseases; manifest lymphatic edema; pregnancy or breastfeeding; or severe psychiatric disorders were regarded ineligible for this trial.

Patients with a long duration of anamnesis and refractory to former treatments could be enrolled. The use of analgesics before and after enrolment was not limited. Patients having undergone surgical interventions or injections to the involved joint after radiotherapy were excluded as soon as this therapy became known.

Prior to enrolment, all patients gave their written informed consent to radiotherapy, participation in this trial, and to the scientific evaluation of the data. The randomization was performed by a statistician (S.G.). Every involved region (knee and/or hand) was counted and randomized separately, so that one to four regions per patient could be treated and analyzed. The joints were assigned to one of the following groups:

- standard-dose group: total dose of 3.0 Gy applied in single fractions of 0.5 Gy twice a week
- experimental-dose group: total dose of 0.3 Gy applied in single fractions of 0.05 Gy twice a week

Radiotherapy sessions were performed Monday/Thursday or Tuesday/Friday to avoid therapy sessions on consecutive days. The dose applied was not known to the patients (single blinded).

Follow-up examinations were scheduled 3 months and 1 year after the end of radiotherapy and were normally performed by examination of the patient in the hospital. In special cases (old age, immobility, reluctance to show up in person), the examination was replaced by a telephone interview. According to our own experience, we choose the length of follow-up taking into account that the vast majority of beneficial effects become apparent after less than 1 year.

Primary endpoints were VAS (visual analogue scale) score, KOOS-PS [8] (knee injury and OA outcome score sum score—physical function short form), SF-SACRAH sum score [9] (short form score for the assessment and quantification of chronic rheumatic affections of the hands), and SF-12 [10] (short form 12, general health status) sum score. Secondary endpoints were SF-12 single scores and the use of analgesic medication.

**Fig. 1:**
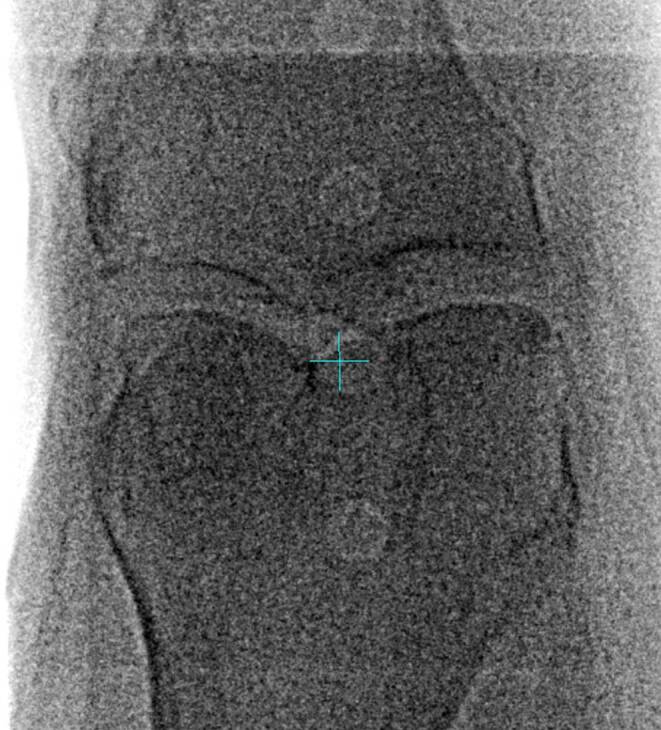
Portal imaging of radiotherapy for osteoarthritis of a knee joint

**Fig. 2:**
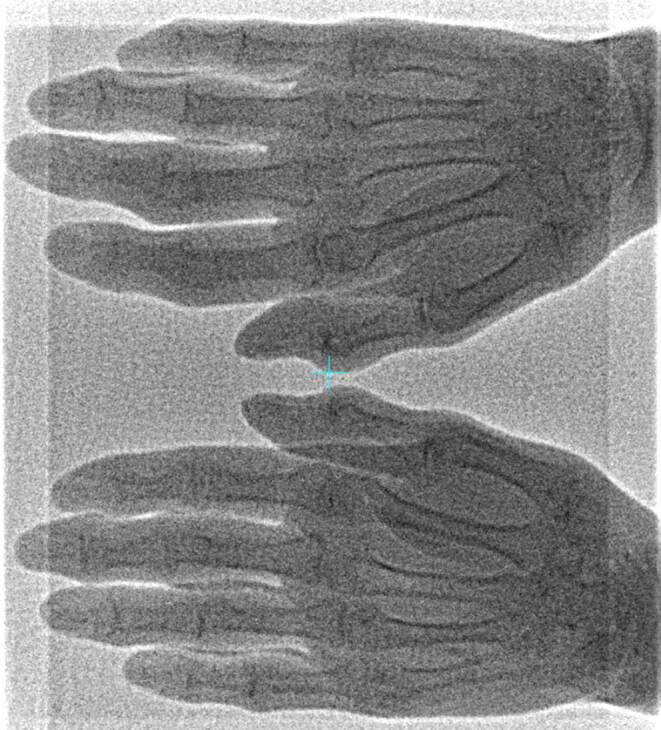
Portal imaging of radiotherapy for osteoarthritis of multiple finger joints

Radiation therapy was applied by a linear accelerator using 6‑MV photons. Knee joints were treated using anteriorly and posteriorly opposing portals. The dose was prescribed to the ICRU reference point in the center of the knee joint. Hand and finger joints were treated by a single dorsal fixed portal, while all involved regions of one hand were included into one planning target volume. The dose was prescribed to the ICRU reference point at the center of the joint. 5 mm thick bolus material was placed above the hand. The dose was calculated individually according to the clinician’s measurements. Figs. 1 and 2 show portal imaging pictures of the radiotherapy for osteoarthritis of the knee and the fingers, respectively.

The trial protocol was approved by the ethics committee of the Saarland physicians’ chamber (no. 60/17 on 19.04.2017). Furthermore, it was approved by the expert committee of the DEGRO (German Society for Radiation Oncology). The research was designed and carried out in agreement with the Declaration of Helsinki in its current version.

To have a power of 90% to detect a difference of 5% in the VAS score with a standard deviation of 12, a total of 135 patients were required cumulatively (knee and hand joints together) in each arm with planned evaluation over 1 year (including a drop-out rate of 10%). Randomization was performed as block randomization. Patients were randomized 1:1 to the treatment arms.

The categorical variables (disease data, see Table 1) were compared using the chi-square test and Fisher’s exact test. Due to a nonnormal distribution, the pain, functional, and quality of life scores in the groups were compared using the Mann–Whitney U test (see Tables 2 and 3). *P*-values ≤ 0.05 were considered as statistically significant. The statistical computations were performed using the MEDLOG™ software package (Fa. Parox, Münster, Germany) after observing the patients for 3 months and were controlled by the statistician. Further details of this trial protocol have been published in the German Clinical Trials Register (DKRS00011870).

**Table 1:** Comparison of patient data

Item	Standard dose group (*n* = 110)	Experimental dose group (*n* = 111)	*p*-value
Mean age (years)	68.2	66.3	t‑test 0.24
*Localization*			
Hand	77 (70%)	81 (73%)	0.06
Knee	33 (30%)	30 (27%)	
Bilateral (patients)	39 (62%)	45 (61%)	0.89
Unilateral (patients)	24 (38%)	29 (39%)	
*Sublocalization (hands)*			
Thumb	18 (23%)	31 (38%)	0.15
Fingers II‑V	10 (13%)	12 (15%)	
Hand joints	2 (3%)	–	
Thumb and other fingers	9 (12%)	8 (10%)	
Thumb and hand joints	2 (3%)	–	
Fingers II–V and hand joints	4 (5%)	1 (1%)	
All	32 (41%)	29 (36%)	
*Mean duration of pain (months)*	56.2	49.6	0.56
SD	52.3	46.0	
*Extension/radiation of pain*			
None	73 (66%)	80 (72%)	0.72
Proximal	22 (20%)	17 (15%)	
Distal	8 (7%)	9 (8%)	
Proximal and distal	7 (7%)	5 (5%)	
*Onset of pain*			
Insidious	93 (85%)	91 (82%)	0.88
Suddenly	12 (11%)	14 (13%)	
Not known	5 (4%)	6 (5%)	
*Impact of pain on quality of life*			
Work	0	2 (2%)	0.37
Leisure	5 (5%)	5 (5%)	
Work and leisure	105 (95%)	104 (93%)	
*Effects on daily work*			
Able to work	71 (64%)	85 (77%)	0.14
Unable to work	34 (31%)	23 (21%)	
No occupation	5 (5%)	3 (2%)	
*Effects on leisure/sports*			
Unlimited	17 (15%)	15 (13%)	0.29
Limited	42 (38%)	54 (49%)	
Impossible	51 (47%)	42 (38%)	
*Therapy before radiotherapy using*			
Ice/heat	52 (47%)	33 (30%)	0.01
Ultrasound	0	0	–
Microwaves	2 (2%)	2 (2%)	0.62
Oral medication	77 (70%)	69 (62%)	0.28
Injections	34 (31%)	24 (22%)	0.16
External splints	4 (4%)	2 (2%)	0.67
Arthroscopy (multiple choices possible)	16 (15%)	12 (11%)	0.53

*SD* standard deviation

**Table 2:** Comparison of pain/function/quality of life data before radiotherapy

Item	Value	Standard dose group (*n* = 110)	Experimental dose group (*n* = 111)	*p*-value
VAS score	*n*	110	110	0.21
	Mean	59.3	57.1	
	SD	16.7	15.0	
	Minimum	10	20	
	Maximum	90	90	
KOOS-PS score (knee joints)	*n*	32	29	0.53
	Mean	20.5	19.9	
	SD	4.9	4.6	
	Minimum	8	8	
	Maximum	28	27	
SF-SACRAH score (hand joints)	*n*	75	80	0.55
	Mean	21.3	20.7	
	SD	10.6	10.4	
	Minimum	3	5	
	Maximum	46	50	
SF-12 somatic doctor	*n* (patients)	68	60	0.06
	Mean	29.8	32.0	
	SD	10.5	9.6	
	Minimum	14	17	
	Maximum	52	52	
SF-12 psychic doctor (patients)	*n* (patients)	68	60	0.15
	Mean	56.0	57.4	
	SD	5.8	7.1	
	Minimum	32	36	
	Maximum	72	73	
SF-12 somatic patient	*n* (patients)	68	60	0.06
	Mean	30.3	33.2	
	SD	11.1	10.0	
	Minimum	15	18	
	Maximum	52	52	
SF-12 psychic patient	*n* (patients)	68	60	0.97
	Mean	57.8	56.7	
	SD	6.7	8.8	
	Minimum	43	29	
	Maximum	72	72	

*SD* standard deviation

**Table 3:** Comparison of pain/function/quality of life data 3 months after radiation therapy to those before radiation therapy

Item (difference of scores 3 months after radiotherapy − scores before radiotherapy)	Value	Standard dose group	Experimental dose group	*p*-value
VAS score	*n*	110	110	0.49
	Mean	−18.9	−15.8	
	SD	27.2	25.5	
	Minimum	−80	−70	
	Maximum	50	60	
KOOS-PS score (knee joints)	*n*	32	29	0.85
	Mean	−5.5	−4.9	
	SD	5.9	5.7	
	Minimum	−19	−15	
	Maximum	7	8	
SF-SACRAH score (hand joints)	*n*	74	80	0.66
	Mean	−5.7	−4.4	
	SD	10.5	10.2	
	Minimum	−38	−32	
	Maximum	7	26	
SF-12 somatic doctor	*n*	67	60	0.19
	Mean	5.7	3.1	
	SD	12.0	10.5	
	Minimum	−25	−18	
	Maximum	36	32	
SF-12 psychic doctor	*n*	67	60	0.42
	Mean	1.2	0.18	
	SD	6.5	7.4	
	Minimum	−16	−18	
	Maximum	23	20	
SF-12 somatic patient	*n*	67	60	0.27
	Mean	5.1	2.8	
	SD	10.2	0.6	
	Minimum	−25	−19	
	Maximum	31	29	
SF-12 psychic patient	*n*	67	60	0.88
	Mean	0.1	0.03	
	SD	6.9	7.6	
	Minimum	−16	−16	
	Maximum	14	21	

*SD* standard deviation

VAS scale: linear scale, 0 = no pain, 100 = maximum imaginable pain, improvement = negative values

KOOS-PS (knee joints): 7 items, 0 = no functional impairment, 100 = maximum impairment, improvement = negative values

SF-SACRAH (hand joints): 7 items, 0 = no functional impairment, 50 = maximum impairment, improvement =negative values

SF-12 scales: 12 items, high values = favorable quality of life, improvement = positive values

## Results

**Fig. 3:**
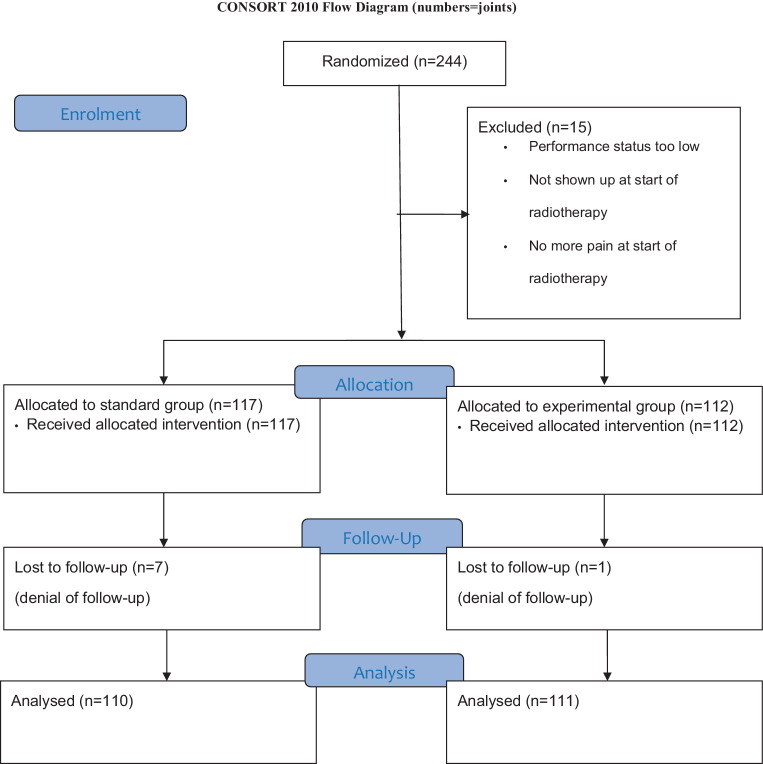
Consort diagram

A total of 244 joints (133 patients) were included in this trial. The majority (220 joints) were included in the University Hospital of Homburg and 24 in the University Hospital of Regensburg. 15 joints had to be excluded due to various reasons (bad health status, pain resolution at the planned start date of radiotherapy, simply not shown up for radiotherapy, see Fig. 3). Of the remaining 229 joints, 117 were assigned to the standard-dose group and the remaining 112 to the experimental-dose group. Of these, 110 joints in the standard-dose group and 111 joints in the experimental-dose group could be followed for at least 3 months.

### Comparison of patient groups before radiotherapy

The mean age at enrolment was 68 years (median 68 years, IQR 19) in the standard-dose group and 66 years (median 65 years, IQR 16) in the experimental-dose group (n. s.). The mean duration of pain anamnesis prior to the start of radiotherapy was 56 months (median 36 months, IQR 72, standard-dose group) and 50 months (median 36 months, IQR 10, experimental-dose group, n. s.). Furthermore, the groups were well balanced with regard to extension and onset of pain, impact of pain on daily life, daily work and leisure, as well as previously applied treatments. There was a trend towards a higher percentage of hand joints in the experimental group (*p* = 0.06) and a significantly higher use of ice treatment in the standard-dose group (*p* = 0.01), which was not regarded to be of clinical significance.

The VAS scores before radiotherapy were not significantly different between the groups (*p* = 0.209). Additionally, the functional scores (KOOS-PS for the knee joints and SF_SACRAH for the hand and finger joints) were not significantly different (*p* = 0.53 and *p* = 0.55, respectively). As to the SF-12 scores, there was a trend in the somatic-doctor score and the somatic-patient score in favor of the experimental-dose group (*p* = 0.06 and *p* = 0.06, respectively).

### Results after 3 months’ follow-up

In summary, we recorded a good analgesic effect of radiotherapy (difference of VAS scores 3 months after vs. those before radiotherapy) in both groups (results in the experimental group in brackets): markedly improved (DeltaVAS ≥ 30 points): 42% (40%), improved (0 < DeltaVAS < 30): 17% (19%), stable 24% (21%), worse 17% (20%). The differences were not statistically significant.

The mean difference in VAS scores after 3 months compared to those before therapy was 18.9 in the standard-dose group and 15.8 in the experimental-dose group (*p* = 0.49). A similar result was achieved for the functional scores (KOOS-PS: *p* = 0.84, SF-SACRAH: *p* = 0.66).

The results concerning quality of life adequately matched those concerning pain and functional impairment. No statistically significant differences could be found (somatic scale, doctor’s judgement: *p* = 0.19; psychic scale, doctor’s judgement: 0.42; somatic scale, patient’s judgement: *p* = 0.27; psychic scale, patient’s judgement: 0.88). No acute side effects were recorded.

Furthermore, there were no statistically significant differences in the results of patients who had a very low pain level after therapy (VAS ≤ 30) compared to those with a higher one.

A subgroup analysis examining the results of the hand joints and the knee joints separately resulted in statistically nonsignificant differences for all scores. Only the VAS score before radiotherapy was found to be a significant prognostic factor for pain relief (univariate search: Spearman and Kedall test, *p* < 0.001), so that patients with a higher pain level before therapy achieved a better result. The location, the duration of anamnesis, and the dose were found to be insignificant. The results of patients with rhizarthrosis compared to those with other locations in the hands were not significantly different. These results were confirmed by multivariate analysis.

## Discussion

The aim of this study was to examine the analgesic effect of the standard dose compared to that of a very low dose. In summary, we found favorable pain relief and a limited improvement in functional and quality of life scores in both arms, there were no statistical differences.

Numerous retrospective studies—some of them even dating back to the 1930s—have shown favorable results concerning pain relief. We are well aware that these trials are of variable quality, the vast majority of the patients having been treated using orthovoltage machines and doses of 6 Gy. The older results have been summarized in the DEGRO (German Society for Radiation Oncology) S2k guideline [11]. To our knowledge, there is only one paper dealing with small joints exclusively, which showed good results as well [12]. More recent retrospective trials have been published by Koc et al. [13], Hautmann et al. [14], Micke et al. [15], and Donaubauer et al. [16]. All of these authors state a significant response of pain to radiotherapy. Hautmann et al. published an additional paper about re-irradiation in patients with insufficient response to the first radiotherapy series or recurrent pain, and regarded a second series as very effective [17]. A systematic review was written by Minten et al. [18]. They summarized that at that time—2016—insufficient data did not allow a valid conclusion to be drawn on the efficacy of radiotherapy.

Two very well designed randomized, controlled, and double-blinded trials were published in 2018 and 2019 (Minten et al. [19] and Mahler et al. [20]), showing no significant benefit for radiotherapy compared to sham therapy. These papers were published during the recruitment for our trial.

We are well aware of the limitations of this trial. This trial needed to be closed prematurely due to slow recruitment of patients. Furthermore, it appears possible that single patients may have guessed their dosage arm, especially when at least two joints in a patient were irradiated with different doses. The influence of oral medication during this trial was not assessed—in our opinion, it was unrealistic to limit intake of the oral analgesics.

These data may give the impression that low doses like 0.3 Gy may be effective in the treatment of inflammatory diseases. Further trials are recommendable: the effect of a conventional dose should be compared to placebo and the effect of low doses should be further investigated. It may be a point of discussion whether only the inflammatory pain component responds to radiotherapy and not the degenerative one. This theory follows the fact that preclinical studies were based on an inflammatory arthritis model rather than an osteoarthritis model [5–7].

## Conclusion

Megavoltage radiotherapy is effective in yielding acceptable pain relief in the majority of patients, with no observed adverse effects. Potentially, very-low-dose radiotherapy is effective as well.
